# The use of near-infrared imaging with indocyanine green in the ovarian tissue transplantation: a case report

**DOI:** 10.52054/FVVO.14.4.051

**Published:** 2023-01-27

**Authors:** D Raimondo, A Raspollini, R Vicenti, F Renzulli, V Magnani, C Franceschini, A Raffone, A Mollo, P Casadio †, R Seracchioli †

**Affiliations:** Division of Gynaecology and Human Reproduction Physiopathology, IRCCS Azienda Ospedaliero-Univeristaria di Bologna, Bologna, Italy; Department of Medical and Surgical Sciences (DIMEC), University of Bologna, Bologna, Italy; Gynecology and Obstetrics Unit, Department of Medicine, Surgery and Dentistry “Schola Medica Salernitana”, University of Salerno, Italy

**Keywords:** Ovarian tissue transplantation, near-infrared intraoperative imaging using indocyanine green, fertility preservation, vascularisation, cryopreservation

## Abstract

The request for fertility preservation has consistently increased in recent years. To our knowledge this case report is the first to describe the application of near-infrared intraoperative imaging using indocyanine green (NIR-ICG) during ovarian tissue transplantation (OTT), to assist surgeon choosing the site of implantation of ovarian fragments. OTT was performed in a 42-year-old woman using NIR-ICG to evaluate the vascularisation of peritoneal area as the site of implantation for the ovarian graft. we believe this new approach could be useful in identifying the best reimplantation site.

## Introduction

The request for fertility preservation for oncological and non-oncological reasons has constantly increased in the last several years (Donnez and Dolmans, 2017). Fertility preservation can be in the form of embryo cryopreservation, mature oocyte cryopreservation after ovarian stimulation and ovarian tissue cryopreservation (OTC) (Practice Committee of the American Society for Reproductive Medicine, 2019; Anderson et al., 2020). Indications for fertility preservation are frequently haematologic and breast cancers. Principal use of chemotherapy, radiotherapy and/or surgery as first line treatment could cause premature ovarian insufficiency (POI) in patients with these pathologies (Donnez and Dolmans, 2013; Wallace et al., 2015; Wallace et al., 2016; Jadoul et al., 2010; Barton et al., 2013; De Vos et al., 2014). Ovarian tissue transplantation (OTT) can be orthotopic (in the pelvic cavity) or heterotopic (outside the pelvic cavity, such as the forearm or the abdominal wall muscle).

A number of studies confirmed that transplanted ovarian tissue, cryopreserved before the gonadotoxic treatment, is effective in terms of both resumption of endocrine function and restoration of fertility. Dolmans et al. (2021) reported 95% of endocrine resumption and 30,6% live birth rate in women who had irregular menses before OTT and 25,4% in women who had amenorrhea before OTT, among 285 women underwent OTT after cancer treatment. These authors also highlighted the importance of finding a well vascularized transplantation site to affix correctly the ovarian tissue and obtain a successful OTT.

Indocyanine green (ICG) is a dye which allows an accurate intraoperative real-time assessment of tissue vascularisation. After an intravenous injection into a peripheral line of 0.2 mg/kg, ICG is excited by near infrared light and emits fluorescence signal detected by dedicated instrumentation (Keller et al., 2017; Diana et al., 2015). This type of enhanced imaging improves the assessment of micro- and macrovascularisation of tissues and organs, allowing an intraoperative real time fluorescence angiography. The use of near-infrared intraoperative imaging using indocyanine green (NIR-ICG) in gynaecological surgery has grown over the last few years; it has been proven safe and useful in several settings such as colorectal and urologic surgery (Raimondo et al., 2020; 2021a; 2021b; Seracchioli et al., 2018; Raimondo et al., 2022a; Vizzielli et al., 2020).

Moreover, NIR-ICG has been reported to be an adequate intraoperative contrast agent with a tissue penetration up to 5 mm. (Raimondo et al., 2020; Alander et al., 2021).

To our knowledge, this case report is the first to describe the application of NIR ICG during OTT, to assist surgeon choosing the site of implantation of ovarian fragments.

## Case Report

In September 2012, a 33-year-old woman was diagnosed with non-Hodgkin marginal zone lymphoma (MZL) stage IVB. The patient was treated with standard regimen R-CHOP (rituximab plus cyclophosphamide, doxorubicin, vincristine, and prednisone) and gonadotrophin releasing hormone analogues.

Several methods for fertility preservation were discussed with the patient, and in agreement with her, laparoscopic ovarian biopsy and ovarian tissue cryopreservation were performed. About 40% of the right ovary was retrieved and the ovarian tissue was cryopreserved according to Fabbri et al. (2000). The retrieval of ovarian tissue was performed only on the right site because the left ovary was mostly occupied by an endometriotic cyst. The postoperative course was uneventful, and thepatient was discharged the day after surgery. In 2015, the patient had amenorrhoea, low anti-Müllerian hormone (<0.02 ug/L) and menopausal levels of follicle stimulating hormone (131.9 U/L), luteinising hormone (65 U/L) and oestradiol (18 pmol/L).

Hormone-replacement therapy was administered. In the following years, the patient remained in complete oncological remission and requested cryopreserved OTT. An assessment of the presence of malignant cells in the cryopreserved ovarian tissue and a gynaecological evaluation were performed. Histological and immunohistochemical analysis of the cryopreserved ovarian tissue revealed no evidence of pathological lymphoid infiltration and showed good preservation of the follicular and stromal component (Fabbri et al., 2018).

**Figure 1 a and b:**
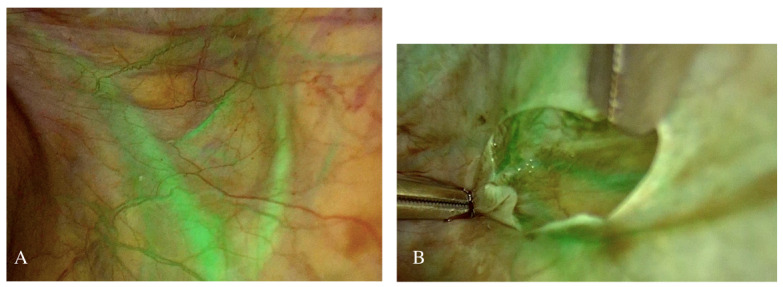
NIR-ICG evaluation of peritoneal area after ovarian tissue transplantation showing adequate vascularization of the transplantation site.

**Figure 2:**
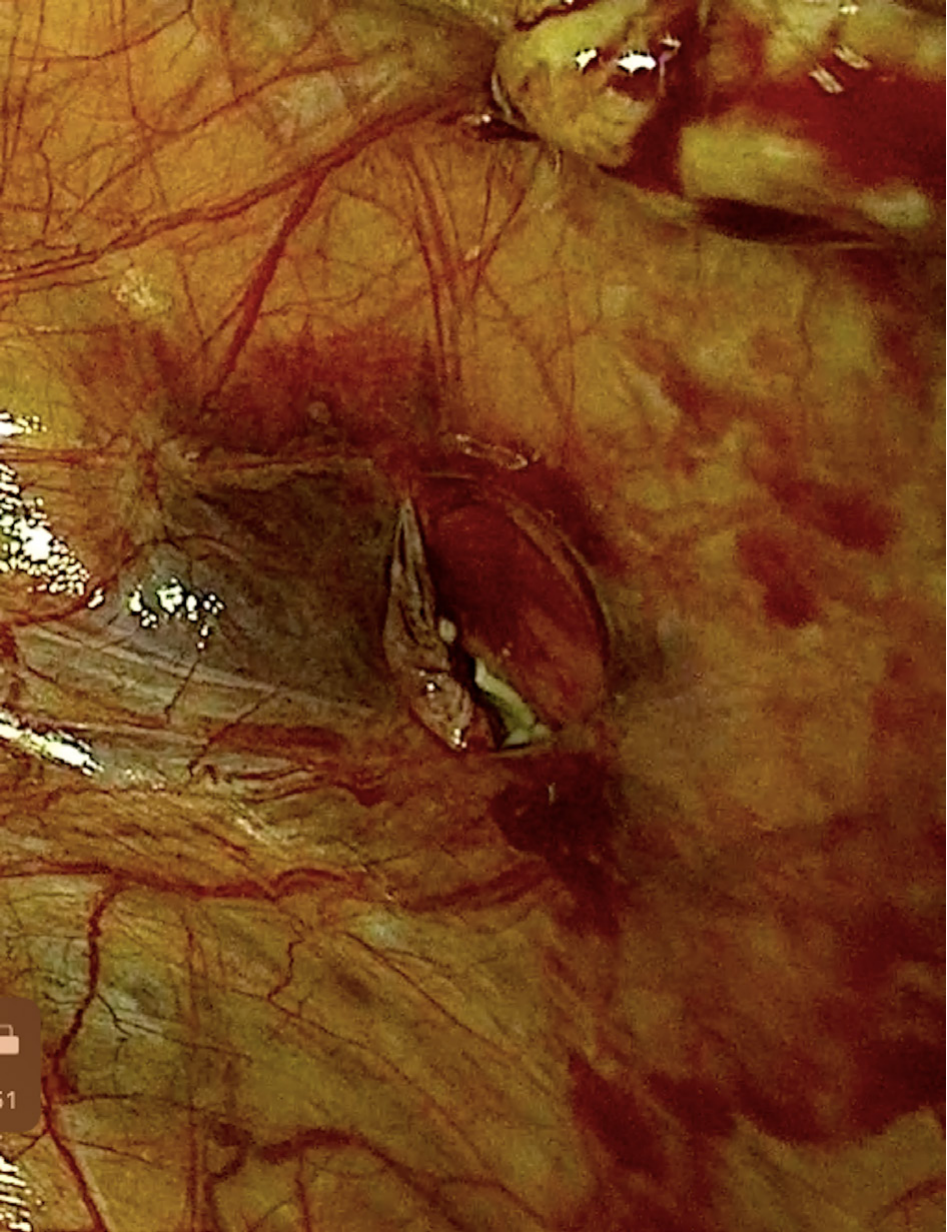
Ovarian fragments in the peritoneal pocket.

In September 2021 a thin endometrial lining (2 mm thickness) and small ovaries with homogeneous structure (right 0.7 cc, left 0.6 cc) were observed on transvaginal ultrasound examination. OTT was planned and an informed consent was obtained before the procedure. During laparoscopy, after ICG injection the most vascularized subperitoneal area of the ovarian fossa was selected using NIR-ICG optic (KARL STORZ, Tuttlingen, Germany) (Figure 1) and 4 cryopreserved fragments were placed in this peritoneal pocket (Fabbri et al., 2014) (Figure 2). Since no consensus exists on the number of individual ovarian pieces needed to improve fertility and/or hormonal activity, we decided to transplant all cryopreserved ovarian tissue (8 samples) given the patient’s age and low ovarian reserve at the time of the transplant. The postoperative course was regular, and the patient was discharged the day after surgery. After 3 months menses returned, and hormonal function was restored.

## Discussion

Advances in diagnosis and introduction of new cancer treatment protocols have significantly improved the survival of young cancer patients. However, these treatments are gonadotoxic: they could induce POI, reducing or destroying the ovarian reproductive and steroidogenic function. From this perspective, fertility preservation has become an important issue to improve quality of life.

A complete reproductive counselling is mandatory not only to choose an appropriate fertility preservation technique but also to take emotional and psychological background into consideration to improve the quality of life of the patients (Vitale et al., 2017; Laganà et al., 2017; Vitale et al., 2018). Ovarian tissue cryopreservation, prior to cancer treatment, is the most feasible alternative to preserve fertility and steroidogenic function in young patients or who cannot delay the start of cancer treatment. At disease remission, cryopreserved tissue can be transplanted, allowing the recovery of ovarian function and spontaneous pregnancy. The efficiency of cryopreserved OTT has been established in terms of ovarian function recovery (95% of cases), number of live births exceeding 200 and induction of puberty (Donnez and Dolmans, 2015; Donnez and Dolmans, 2017; Donnez et al., 2015; Raimondo et al., 2022b).

Despite these encouraging results, further progress is necessary to improve the ovarian graft activity and the outcomes of OTT. In particular, it is important to develop new cryopreservation strategies in order to minimise tissue damage, both during and after OTT. Of note, avascular grafting technique and potential ischaemic tissue damage may lead to apoptosis and loss of primordial follicles up to 90% in the first few days and fibrotic changes. Neovascularisation of ovarian tissue graft starts 2–3 days after transplantation in animal models, with reoxygenation taking place 7 to 10 days after OTT (Li et al., 2016). As the outcomes of transplanted frozen thawed ovarian tissue depend on graft ischaemia, further studies are necessary to implement a technology able to identify well vascularised retroperitoneal area and provide an adequate and prompt revascularisation.

NIR-ICG has been proven as a reliable technology to discriminate between ischaemic/ hypoxic areas from regular ones in several experimental studies (Duprée et al., 2020). Several studies described feasibility, safety and efficacy of fluorescence angiography using intravenous ICG to assess tissue real-time vascularisation of pelvic organs (e.g., bowel, ureter, and vagina) and peritoneum (Raimondo et al.,2021a; Vizzielli et al., 2020; Mandrova et al., 2019; Cosentino et al., 2018), making this technology a potential valuable method for OTT (Raimondo et al., 2020; Alander et al., 2012).

To the best of our knowledge, this is the first case report showing a novel use of NIR-ICG to select the most vascularised retroperitoneal site of implantation of the ovarian graft in the pelvis.

## Conclusion

ICG fluorescence imaging is a potential valuable technology to assess intraoperatively the vascularisation of subperitoneal tissue and select the most vascularised area as transplantation site. NIR-ICG could reduce the hypoxic-ischaemic damage to which ovarian tissue are subjected in the first days post-transplant improving reproductive possibilities and prolonged steroidogenic activity. Future studies are needed to prove potential benefits of this technology during OTT.
